# Pregnancy-associated plasma protein-A is a stronger predictor for adverse cardiovascular outcomes after acute coronary syndrome in type-2 diabetes mellitus

**DOI:** 10.1186/s12933-017-0526-6

**Published:** 2017-04-05

**Authors:** Wei-Ping Li, Moni B. Neradilek, Fu-Sheng Gu, Daniel A. Isquith, Zhi-Jun Sun, Xing Wu, Hong-Wei Li, Xue-Qiao Zhao

**Affiliations:** 1 Cardiovascular Center, Beijing Friendship Hospital, Capital Medical University, Beijing, China; 2 Key Laboratory, Metabolic Disorders Related Cardiovascular Disease, Beijing, China; 3 The Mountain-Whisper-Light Statistics, Seattle, WA USA; 4 Clinical Atherosclerosis Research Lab, Division of Cardiology, University of Washington, Seattle, WA USA

**Keywords:** Type-2 diabetes mellitus, Acute coronary syndrome, Pregnancy-associated plasma protein-A, Cardiovascular outcomes

## Abstract

**Background:**

The risk prediction of pregnancy-associated plasma protein-A (PAPP-A) for future cardiovascular (CV) events post acute coronary syndrome (ACS) in patients with type-2 diabetes mellitus (T2DM) was investigated in comparison to other risk factors.

**Methods:**

PAPP-A was measured at hospital admission in 320 consecutive ACS patients (136 with T2DM and 184 without). All patients were followed for 2 years for occurrence of CV death, non-fatal MI or stroke. Effect of PAPP-A on the CV event risk was estimated using Cox regression models. Receiver operating characteristics (ROC) curves were generated to demonstrate the sensitivity and specificity of PAPP-A in predicting CV events.

**Results:**

ACS patients with T2DM had higher PAPP-A (19.29 ± 16.36 vs. 13.29 ± 13.90 ng/ml, p < 0.001) and higher rate of CV events 2 years post ACS (27.2 vs. 13.6%, p = 0.002) than those without. Higher levels of PAPP-A were significantly associated with increased risk of CV events during 2-year follow-up [HR = 2.97 for 1 SD increase in log_10_(PAPP-A), 95% CI 2.11–4.18, p < 0.001] in T2DM and (HR = 3.16, 95% CI 2.27–4.39, p < 0.001) in non-T2DM. Among patients with T2DM, PAPP-A showed a larger area under the curve (AUC 0.79) that was significantly more predictive than hsCRP (AUC 0.64), eGFR (AUC 0.66) and LVEF < 50% (AUC 0.52); predictive ability did not improve significantly by including those factors into the model.

**Conclusions:**

Patients with T2DM had higher levels of PAPP-A and increased risk of CV events. Elevated PAPP-A compared to other risk factors was a stronger predictor for future CV events 2 years post ACS in patients with T2DM.

*Trial registration* ISRCTN10805074. Registered on 20 January 2017, retrospectively registered.

## Background

Patients with type-2 diabetes mellitus (T2DM) are known to suffer worse outcomes post-acute coronary syndrome (ACS) [1, 2]. Identification of factors that can improve the risk prediction of cardiovascular outcomes in T2DM remains clinically challenging [3]. Previous research has found that pregnancy-associated plasma protein-A (PAPP-A), a high molecular weight and zinc-binding metalloproteinase, is an important regulatory protein in cell proliferation and the development of atherosclerosis and can degrade the proteins that maintain the integrity of the protective fibrous cap of atherosclerotic plaques [4, 5]. PAPP-A, originally found in pregnant women, is produced by syncytiotrophoblast cells in a heterotetrameric complex (500 kDa) [6], but also by osteoblasts, fibroblasts, endothelial cells, vascular smooth muscle cells [4, 7, 8] and by monocytes and macrophages [9, 10] in a homodimer (400 kDa) with proteolytic activity [6]. Previous studies have demonstrated that PAPP-A is a potentially important biomarker of plaque instability and inflammation in patients with ACS [11, 12]. PAPP-A has been identified in vulnerable coronary plaques but not found in stable plaques [13] and higher PAPP-A levels are associated with higher 3-vessel thin-cap fibroatheroma (TCFA) burden in patients with coronary artery disease [14]. Furthermore, subjects with T2DM appeared to have higher levels of PAPP-A than age-matched controls [15, 16]. However, whether the increased levels of PAPP-A in T2DM can better predict future CV events post ACS had not been studied. Therefore, we prospectively investigated the risk prediction of serum PAPP-A concentrations for future CV events in ACS patients with and without T2DM in comparison to other risk factors.

## Methods

### Study population

**Fig. 1:**
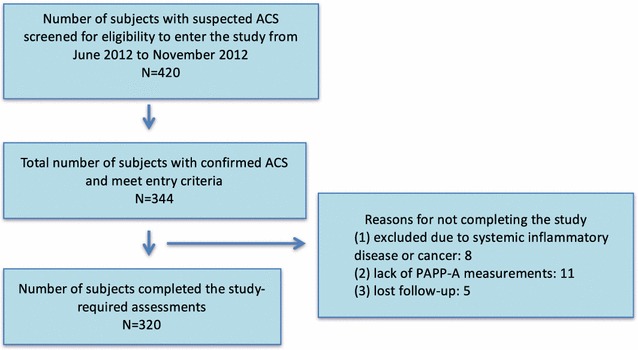
Study subject enrollment and completion

As shown in Fig. 1, a total of 420 patients with suspected ACS including ST-elevation myocardial infarction (STEMI), non-ST-elevation MI (Non-STEMI) and unstable angina (UA) were admitted to CCU and 1 of 4 cardiac wards at the Beijing Friendship Hospital Cardiovascular Center between June and November 2012. Of these 420 patients, 344 were confirmed with ACS [17, 18]. Three hundred twenty subjects completed the study and were included in the final analysis.

Informed consent was obtained from all subjects prior to any study related procedures or measurements. The study protocol and procedures received approval from the Beijing Friendship Hospital Institutional Review Board and were performed to conform to the Declaration of Helsinki.

T2DM was diagnosed based on fasting plasma glucose ≥7.0 mmol/l, or 2-h plasma glucose ≥11.1 mmol/l, or HbA1C ≥6.5%, or receiving treatment for diabetes [19].

### Laboratory analysis

Routine laboratory tests on lipids, glucose level, Hs-CRP, hepatic and renal functions were performed using the standardized methods at the Beijing Friendship Hospital Laboratory. Cardiac troponin I (cTnI) was detected by electro-chemiluminescence technology for quantitative measurement (3rd generation TnI, Elecsys 2010, Roche, Mannheim, Germany). The lower detection limit is 0.01 µg/l with a recommended diagnostic threshold of 0.03 µg/l for MI. N-terminal B-type natriuretic peptide (NT-proBNP) was measured using electrochemiluminescence immunoassays performed on a Roche Elecsys 2010 automated platform (Roche Diagnostics, Burgess Hill, UK). Additional blood samples were collected with EDTA preparation at the time of routine laboratory tests [20] and were stored at −80 °C.

Total serum PAPP-A was measured before heparin administration and blinded to subject characteristics, laboratory results and clinical course using the ultra-sensitive enzyme-linked immunosorbent assay (ELISA) method with kits manufactured by DRG Instruments GmBH (Germany). This method with sensitivity of 0.023 ng/ml is specially designed to detect low concentrations of circulating enzyme that is associated with possible plaque rupture [21].

### Echocardiograms

Echocardiography was performed routinely at hospital admission. Digital echocardiograms were analyzed on the EchoPAC workstation (Philips IE Elite). Left ventricular end-diastolic and -systolic volumes were obtained using Simpson’s method of discs in the apical 4- and 2-chamber views as recommended by the American Society of Echocardiography [22]. Left ventricular ejection fraction (LVEF) was calculated using left ventricular volumes and the formula: [(end-diastolic volume − end-systolic volume)/end-diastolic volume] × 100%.

### CV outcomes

Events including CV death, nonfatal MI or stroke were identified during the initial hospitalization for ACS, and at 6 month intervals for up to 24 months per the study follow-up schedule. Each event was evaluated and confirmed with medical records. Events possibly related to ACS treatment procedures (PCI or CABG) were excluded. A composite of CV death, nonfatal MI or stroke was the pre-defined study primary endpoint.

### Statistical analysis

Comparisons of clinical characteristics and laboratory measurements between subjects with and without T2DM were performed using unpaired t-tests for continuous variables and Chi square tests for categorical variables. Kaplan–Meier curves and Cox regression for the composite CV event were generated to compare the event-free survival difference between subjects with or without T2DM.

Cox regression analysis was used to determine the factors that were associated with the risk of subsequent CV events post-ACS. Four models were considered for the effect of PAPP-A on the risk of events: (1) model with PAPP-A value, (2) model with log_10_(PAPP-A), (3) model with linear spline for PAPP-A (with a knot at the 60th percentile, PAPPA = 13.16 ng/ml) and (4) categorical model (lower quartile ≤8.46 ng/ml, 2nd and 3rd quartiles combined >8.46 and <17.3 ng/ml and 4th quartile ≥17.4 ng/ml). Among the four candidate models log_10_(PAPP-A) was selected for the largest Cox & Snell pseudo R^2^ [23].

The effect of PAPP-A on the risk of CV events was also tested in multivariate models that adjusted for covariates including: diabetes status, age, gender, LVEF (%), HbA1C, Troponin I and hsCRP. Due to the limited number of events (37 events in T2DM subjects and 24 in non-T2DM subjects) only a single covariate was adjusted for at a time. A covariate was considered as confounding if the adjustment for the covariate changed the hazard ratio (HR) for PAPPA-A by at least 10%.

Receiver operating characteristics (ROC) curves were generated to demonstrate the ability to predict CV events. Area under the ROC curve (AUCs) was calculated for single predictor and selected multivariate models (the latter based on logistic regression). The multivariate logistic regression models used all predictors that had AUC > 0.6. To determine whether PAPP-A had a different ability to predict CV events compared to other single factors or to the multivariate model, we used DeLong’s test to compare pairs of correlated ROC curves [24]. We would like to point out that this test is perhaps unfair to PAPP-A when it is being compared to the multivariate model because PAPP-A is part of the multivariate model and DeLong’s test does not account for the number of other predictors in the multivariate model. Confidence intervals for AUC were calculated with the non-parametric bootstrap with 2000 replicates.

All tests were two tailed and p < 0.05 was considered statistically significant. All statistical analyses were performed using R version 3.1.1 (R Foundation for Statistical Computing, Vienna, Austria).

## Results

### Clinical and laboratory characteristics

Overall, among the 320 subjects, 68% were male, mean age was 65 years (range 27–90 years), 31% were diagnosed with STEMI, 26% with non-ST elevation MI and 43% with unstable angina. Of the 320 subjects, 168 (53%) received PCI, 11 (3%) received CABG in addition to medical management, and 141 (44%) were treated only with medical therapy during hospitalization. The proportion of medical management during hospitalization and at discharge was ASA (97%), P2Y_12_ inhibitor (72%), statin (92%), β-blocker (75%) and angiotensin converting enzyme inhibitor/angiotensin-receptor blocker (72%). There was no significant difference in treatment during hospitalization between subjects with and without T2DM (Table 1).

**Table 1:** Baseline clinical characteristics and laboratory assessment

	All (n = 320)	T2DM (−) (n = 184)	T2DM (+) (n = 136)	p value
Age, years	65 ± 12	64 ± 12	66 ± 11	0.108
Male, n (%)	218 (68)	135 (73)	83 (61)	0.021
Physical sign				
Body mass index, kg/m^2^	25.4 ± 3.5	25.3 ± 3.4	25.5 ± 3.6	0.661
Systolic BP, mmHg	129 ± 21	128 ± 19	132 ± 24	0.090
Diastolic BP, mmHg	74 ± 12	74 ± 12	74 ± 12	0.078
Cardiovascular risk factors				
Current smoking	117 (37)	73 (40)	44 (32)	0.179
History of smoking	146 (46)	91 (50)	55 (40)	0.114
Hypertension	205 (64)	112 (61)	93 (68)	0.195
Hyperlipidemia	174 (54)	91 (50)	83 (61)	0.042
Previous CAD	72 (23)	44 (24)	28 (21)	0.502
Previous MI	39 (12)	21 (11)	18 (13)	0.730
Previous PCI or CABG	59 (18)	34 (19)	25 (18)	1.000
Previous stroke	43 (13)	24 (13)	19 (14)	0.869
ACS diagnosis and treatment				
ST elevation MI	101 (31)	58 (31)	43 (31)	0.985
Non-ST elevation MI	82 (26)	47 (26)	35 (26)	0.969
Unstable angina	137 (43)	79 (43)	58 (43)	0.959
PCI	168 (53)	94 (51)	74 (54)	0.556
CABG	11 (3)	3 (2)	8 (6)	0.080
Medical therapy				
ASA	309 (97)	180 (98)	129 (95)	0.257
Y2P_12_ inhibitor	231 (72)	132 (72)	99 (73)	0.835
Statin	293 (92)	170 (92)	123 (90)	0.535
β-Blocker	240 (75)	133 (72)	107 (79)	0.192
ACEI/ARBs	229 (72)	129 (70)	100 (74)	0.503
LVEF, %	59 ± 12	60 ± 12	56 ± 12	0.001
Laboratory assessment				
Fasting glucose, mmol/l	6.6 ± 3.0	5.5 ± 1.7	8.2 ± 3.5	<0.001
HbA1C, %	6.5 ± 1.5	5.7 ± 0.5	7.6 ± 1.8	<0.001
Total cholesterol, mmol/l	4.3 ± 1.0	4.2 ± 1.0	4.4 ± 1.1	0.322
Triglycerides, mmol/l	1.85 ± 1.21	1.65 ± 1.04	2.13 ± 1.37	<0.001
LDL-C, mmol/l	2.24 ± 0.59	2.23 ± 0.59	2.24 ± 0.59	0.937
HDL-C, mmol/l	0.93 ± 0.22	0.94 ± 0.21	0.92 ± 0.23	0.229
AST, U/L	68.2 ± 94.6	67.1 ± 83.9	69.7 ± 107.7	0.333
ALT, U/L	28.2 ± 24.9	27.4 ± 22.8	29.2 ± 27.5	0.607
Creatinine µmol/l	102.3 ± 117.4	101.5 ± 118.5	103.4 ± 116.6	0.961
hsCRP, ng/ml	7.3 ± 5.8	6.5 ± 5.7	8.4 ± 5.9	<0.001
PAPP-A, ng/ml	15.8 ± 15.3	13.3 ± 13.9	19.3 ± 16.4	<0.001
CK-MB, ng/ml	14.8 ± 37.1	17.9 ± 44.7	10.6 ± 22.8	0.713
Troponin I, ng/ml	3.3 ± 8.4	3.2 ± 8.3	3.5 ± 8.4	0.474
NT-proBNP, pg/ml	2352.5 ± 5746.6	1745.8 ± 4603.3	3173.4 ± 6937.3	0.128
eGFR, ml/min 1.73 m^2^	81.3 ± 23.4	82.7 ± 21.6	79.4 ± 25.6	0.217

Values are mean ± SD or n (%)

*T2DM* type-2 diabetes mellitus, *BP* blood pressure, *MI* myocardial infarction, *CAD* coronary artery disease, *PCI* percutaneous coronary intervention, *CABG* coronary artery bypass graft, *ACEI* angiotensin converting enzyme inhibitor, *ARB* angiotensin-receptor blocker, *LVEF* left ventricular ejection fraction, *LDL-C* low density lipoprotein cholesterol, *HDL-C* high density lipoprotein cholesterol, *AST* aspartate aminotransferase, *ALT* alanine aminotransferase, *hsCRP* high sensitive C-reactive protein, *PAPP-A* pregnancy-associated plasma protein-A, *NT-proBNP* N-terminal B-type natriuretic peptide, *eGFR* estimated glomerular filtration rate

As shown in Table 1, 136 subjects with T2DM, compared to 184 without, were significantly more likely to be female (39 vs. 27%, p = 0.021), to have hyperlipidemia (61 vs. 49%, p = 0.042) and lower LVEF (56 ± 12 vs. 60 ± 12%, p = 0.001). Subjects with T2DM had significantly higher PAPP-A concentrations (19.3 ± 16.4 vs. 13.3 ± 13.9 ng/ml, p < 0.001) and hsCRP levels (8.4 ± 5.9 vs. 6.5 ± 5.7 ng/ml, p < 0.001) than those without. As expected, subjects with T2DM showed significantly higher levels of fasting blood glucose (8.2 ± 3.5 vs. 5.5 ± 1.7 mmol/l, p < 0.001) and HbA1C (7.6 ± 1.8 vs. 5.7 ± 0.5%, p < 0.001). The T2DM group also had higher triglycerides (2.13 ± 1.37 vs. 1.65 ± 1.04 mmol/l, p < 0.001), but no significant difference was seen in the levels of total cholesterol, low-density lipoprotein cholesterol (LDL-C), high-density lipoprotein cholesterol (HDL-C), aspartate aminotransferase (AST), alanine aminotransferase (ALT), creatinine, CK-MB, cTnI, NT-proBNP or eGFR.

### CV outcomes

**Fig. 2:**
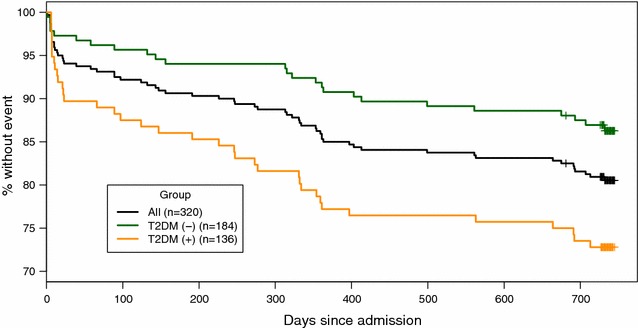
Cardiovascular outcomes during 2 years post ACS. Kaplan–Meier estimated event-free survival for the composite cardiovascular outcome (composite of cardiovascular death, non-fatal myocardial infarction or non-fatal ischemic stroke) in all ACS patients and patients with or without T2DM

During the 24 months of follow-up, 4.4, 9.0 and 7.8% were confirmed for CV death, nonfatal MI and stroke, respectively. Overall, there were 62 patients (37 with T2DM and 25 without T2DM) with a CV event. The overall 2-year event free survival rate for the composite CV outcome was 80.9% (95% CI 76.7, 85.4). As shown in Fig. 2, the 2-year event free survival rate in subjects with T2DM was 72.8% (95% CI 65.7–80.7), which was, not surprisingly, significantly lower than that in subjects without T2DM 86.9% (95% CI 82.2–92.0) (p = 0.002, Cox regression).

### Unadjusted and adjusted association between PAPP-A and CV outcomes

The univariate Cox regression analysis identified that elevated PAPP-A was significantly associated with increased occurrence of CV events in all subjects and subjects with and without T2DM as shown in Table 2. HR per 1 SD increase in log_10_(PAPP-A) was 2.97 (95% CI 2.11–4.18) in subjects with T2DM, 3.16 (95% CI 2.27–4.39) in those without T2DM and 3.10 (95% CI 2.48–3.87) in all subjects, all p < 0.001, respectively. In addition, older age, hypertension, lower LVEF, higher HbA1C, lower eGFR, higher cTnT and higher hsCRP were also significantly associated with increased risk of CV events in the univariate analysis.

**Table 2:** Univariate Cox regression analysis for cardiovascular outcome

	All (n = 320)		T2DM (−) (n = 184)		T2DM (+) (n = 136)	
	HR (95% CI)	p value	HR (95% CI)	p value	HR (95% CI)	p value
Age, per 1SD	1.65 (1.26–2.17)	<0.001	1.42 (0.94–2.15)	0.09	1.82 (1.25–2.63)	0.002
Male gender	1.45 (0.87–2.41)	0.2	0.69 (0.26–1.84)	0.5	1.85 (0.97–3.53)	0.06
BMI, per 1SD	0.84 (0.65–1.09)	0.2	0.82 (0.55–1.23)	0.3	0.84 (0.60–1.18)	0.3
Hypertension	2.04 (1.12–3.70)	0.02	2.75 (1.03–7.33)	0.04	1.47 (0.69–3.11)	0.3
Hyperlipidemia	0.81 (0.49–1.34)	0.4	0.76 (0.34–1.67)	0.5	0.74 (0.39–1.40)	0.4
Previous CAD	1.54 (0.90–2.64)	0.12	2.19 (0.98–4.88)	0.055	1.24 (0.59–2.64)	0.6
Previous stroke	1.07 (0.53–2.16)	0.9	1.62 (0.61–4.31)	0.3	0.74 (0.26–2.09)	0.6
LVEF (%), per 1SD	0.70 (0.55–0.88)	0.003	0.61 (0.43–0.86)	0.005	0.87 (0.63–1.20)	0.4
HbA1C, per 1SD	1.32 (1.06–1.63)	0.01	0.82 (0.51–1.31)	0.4	1.14 (0.82–1.57)	0.4
log_10_(TG), per 1SD	0.92 (0.72–1.19)	0.5	0.89 (0.60–1.33)	0.6	0.82 (0.58–1.15)	0.2
TC, per 1SD	0.87 (0.67–1.13)	0.3	0.94 (0.62–1.42)	0.8	0.80 (0.58–1.11)	0.2
HDL-C, per 1SD	0.95 (0.73–1.23)	0.7	0.81 (0.52–1.27)	0.4	1.05 (0.77–1.44)	0.8
LDL-C, per 1SD	0.88 (0.68–1.14)	0.3	1.06 (0.71–1.57)	0.8	0.77 (0.56–1.07)	0.12
GFR, per 1SD	0.60 (0.48–0.74)	<0.001	0.65 (0.47–0.90)	0.008	0.57 (0.42–0.77)	<0.001
CK-MB, per 1SD	1.01 (0.79–1.31)	0.9	1.16 (0.86–1.57)	0.3	0.85 (0.56–1.31)	0.5
Troponin I, per 1SD	1.30 (1.10–1.54)	0.002	1.23 (0.92–1.66)	0.2	1.33 (1.10–1.62)	0.004
hsCRP, per 1SD	1.84 (1.43–2.37)	<0.001	1.77 (1.20–2.60)	0.004	1.75 (1.25–2.46)	0.001
log_10_(PAPP-A), per 1SD	3.10 (2.48–3.87)	<0.001	3.16 (2.27–4.39)	<0.001	2.97 (2.11–4.18)	<0.001

*HR* hazard ratio, *CI* confidence interval, *LVEF (%)* percentage of LVEF >50%, other abbreviations as in Table 1

After the adjustment for age, gender, LVEF, HbA1C, cTnI or hsCRP (a single adjustment variable at a time), the effect of PAPP-A on CV events in all subjects and subjects with and without T2DM changed by <10% suggesting that these factors do not confound the relationship between PAPP-A and the CV event risk (Table 3).

**Table 3:** Unadjusted and adjusted effects of PAPP-A

	All (n = 320)		T2DM (−) (n = 184)		T2DM (+) (n = 136)	
	HR (95% CI)*	% Δ** (%)	HR (95% CI)*	% Δ** (%)	HR (95% CI)*	%Δ** (%)
Unadjusted	3.10 (2.48–3.87)	0	3.16 (2.27–4.39)	0	2.97 (2.11–4.18)	0
Adjusted for						
Age	2.93 (2.34–3.66)	−5	3.04 (2.14–4.32)	−4	3.16 (2.21–4.53)	6
Sex	3.05 (2.44–3.82)	−1	3.36 (2.36–4.79)	7	3.04 (2.14–4.31)	2
LVEF (%)	3.02 (2.41–3.79)	−3	2.96 (2.11–4.17)	−6	2.97 (2.11–4.19)	0
HbA1C	3.06 (2.43–3.86)	−1	3.18 (2.23–4.55)	1	2.98 (2.11–4.19)	0
Troponin I	3.09 (2.46–3.88)	0	3.18 (2.27–4.45)	1	2.89 (2.05–4.07)	−3
hsCRP	2.86 (2.21–3.69)	−8	2.95 (2.02–4.30)	−7	2.79 (1.88–4.13)	−6

HR for 1 SD increase in log_10_ PAPP-A for single covariates. All adjusted HRs for PAPP-A are adjusted for the single variable that is specified

* p < 0.001 for all variables

** Percent change in HR compared to the unadjusted HR

### Predictive effects of PAPP-A on CV outcomes

**Fig. 3:**
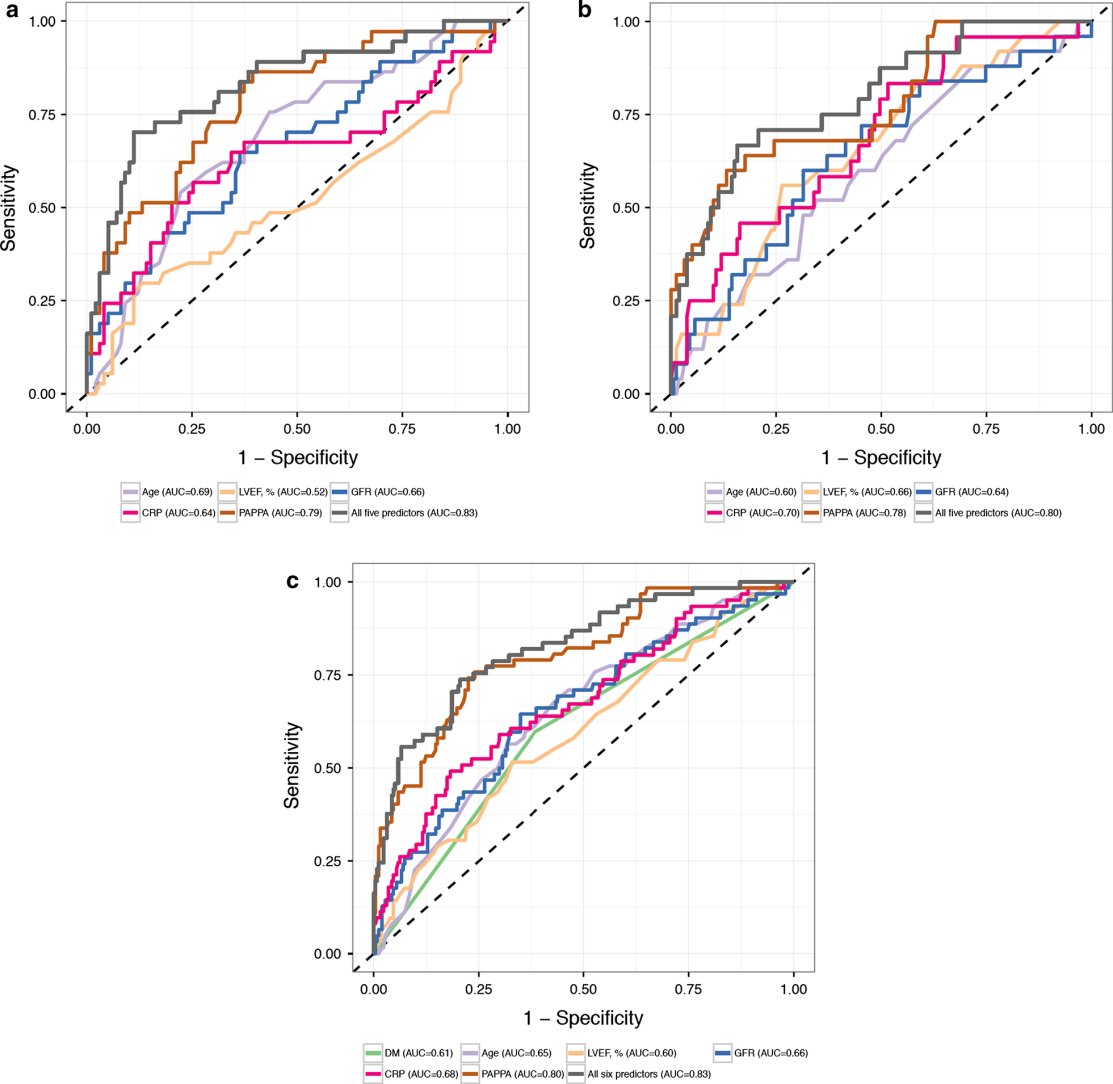
Predictors of cardiovascular outcome 2 years post ACS. Receiver operating characteristics (ROC) curves for PAPP-A and other factors predicting cardiovascular outcome 2 years post ACS in **a** patients with T2DM; **b** patients without T2DM; and **c** all patients. Specifically, in ACS patients with T2DM, area under the ROC curve (AUC) was 0.79 for PAPP-A in *brown color line*, 0.69 for age in *purple*, 0.52 for LVEF in *gold*, 0.66 for GFR in *blue*, 0.64 for hsCRP in *red*, and 0.83 for all 5 factors combined in *black*

Areas under the ROC curves (AUC) for PAPP-A, age, LVEF, eGFR, hsCRP and the combination of all these predictors (from a multivariate logistic regression including all predictors) are shown in Fig. 3 and Table 4. Specifically, among subjects with T2DM (Fig. 3a), PAPP-A showed AUC of 0.79 (95% CI 0.70–0.87) which was significantly larger than LVEF (AUC 0.52; 95% CI 0.40–0.64; p < 0.001), eGFR (AUC 0.66; 95% CI 0.56–0.77; p = 0.03) and hsCRP (AUC 0.64; 95% CI 0.52–0.76; p = 0.01). Similar results were also seen in those without T2DM (Fig. 3b) and in all subjects (Fig. 3c).

**Table 4:** Area under the ROC curve (AUC) for PAPP-A, other selected factors and a multivariate model (all factors)

	All (n = 320)		T2DM (−) (n = 184)		T2DM (+) (n = 136)	
	AUC (95% CI)	p value	AUC (95% CI)	p value	AUC (95% CI)	p value
PAPP-A	0.80 (0.74–0.87)	Ref.	0.78 (0.67–0.88)	Ref.	0.79 (0.70–0.87)	Ref.
Diabetes	0.61 (0.54–0.67)	<0.001				
Age	0.65 (0.58–0.73)	0.003	0.60 (0.48–0.72)	0.051	0.69 (0.59–0.79)	0.15
LVEF (%)	0.60 (0.52–0.68)	<0.001	0.66 (0.54–0.77)	0.06	0.52 (0.40–0.64)	<0.001
eGFR	0.66 (0.58–0.74)	<0.001	0.64 (0.52–0.76)	0.051	0.66 (0.56–0.77)	0.03
hsCRP	0.68 (0.60–0.75)	0.003	0.69 (0.57–0.80)	0.2	0.64 (0.52–0.76)	0.01
Multivariate (all above)	0.83 (0.77–0.89)	0.12	0.80 (0.70–0.90)	0.2	0.83 (0.75–0.91)	0.051

*AUC* areas under the receiver operating characteristics curve, *Multivariate* a multivariate logistic regression using all factors presented in this table, *p value* comparison to the PAPP-A model; *Ref*. reference

Furthermore, as shown in Table 4, when PAPP-A was combined with other factors, the AUC improved to 0.83 (95% CI 0.75–0.91) in subjects with T2DM, 0.83 (95% CI 0.77–0.89) in all subjects, and 0.80 (95% CI 0.70–0.90) in those without T2DM. None of these improvements in AUC was statistically significant compared to PAPP-A alone.

## Discussion

### Recurrent CV events post ACS in T2DM

Recurrent cardiovascular events in post ACS patients treated with current standard medical therapy and revascularization interventions (PCI or CABG) remain an important clinical issue [25, 26]. A multinational study by the GRACE investigators [27] showed that CV death occurred in 4.1%, re-infarction in 4.4%, and stroke in 1.3% during 2 years of follow-up in 22,937 ACS patients from 57 sites. Our study found 4.4% CV death, 9.0% nonfatal MI and 7.8% stroke during 24 months of post ACS. The higher recurrent event rates in our study could be associated with a higher percentage of patients with T2DM (43%) compared to 25% in GRACE since other patient characteristics and ACS treatment were similar between our study and GRACE [27]. Indeed, we found a higher composite endpoint (CV death, nonfatal MI, stroke) rate during 24 months post ACS in the patients with T2DM than those without (27 vs. 13%), which is consistent with the accumulated evidence that diabetes increases the risk of recurrent cardiovascular events post ACS [28–30].

### PAPP-A forms and levels among populations

There are 2 forms of PAPP-A. During pregnancy, PAPP-A produced by placental syncytiotrophoblast cells is a heterotetrameric complex (500 kDa) covalently linked with eosinophil major basic protein [6]. This form is proteolytically inactive. The distribution of PAPP-A was investigated in pregnant women and found that it varies by ethnicity, gestational age, maternal weight and smoking status [31–35]. The clinical investigations of the complex relationship among PAPP-A levels during pregnancy, gestational diabetes (GDM) and cardiovascular disease risk are evolving [36–39]. Low levels of PAPP-A during the first trimester are associated with fetal Down’s syndrome [40] and other adverse events [37, 41–43]. A recent study [39] among Chinese women showed that PAPP-A levels in the first trimester were not predictive of development of GDM which is highly prevalent (approximately 19%) in China [44]. History of GDM in the 102 women (100 post-menopause) enrolled in our study was not collected.

In contrast, the other form of PAPP-A secreted by vascular cells [4, 7–10] is a homodimer (400 kDa) not covalently linked with eosinophil major basic protein. This form has proteolytic activity [6] and is considered to play an important role in cardiovascular disease [45]. In general, the concentration of PAPP-A is found to be very low in adult males and non-pregnant females [16]. In addition to the association of elevated PAPP-A levels with atherosclerotic vascular disease [11–14], recently, PAPP-A was found to be a useful biomarker for cardiovascular dysfunction, inflammatory state and malnutrition in chronic kidney disease (CKD) patients undergoing hemodialysis [46]. Additionally, PAPP-A may be involved in the pathogenesis of retinal vein occlusion (RVD) [47] and the development of chronic obstructive pulmonary disease (COPD) [48].

PAPP-A was reported to be higher in T2DM than healthy controls in 2 studies [15, 16], appeared to be negatively associated with HbA1C levels [49], and its expression in glomeruli was associated with diabetic nephropathy [50]. Our study found significantly higher concentrations of PAPP-A in ACS patients with T2DM than those without (19 vs. 13 ng/ml, p < 0.001). These results represent the increased inflammatory state in ACS and T2DM, particularly in ACS combined with T2DM [51] and is supported by previous investigations [9, 10, 13, 14]. These investigations have demonstrated that PAPP-A was produced and released by monocytes/macrophages, was found in ruptured and eroded plaques and co-localized with vascular smooth muscle cells and activated macrophages, and was associated with higher 3-vessel TCFA burden as assessed by virtual histology (VH)-intravascular ultrasound (IVUS) [14] and carotid plaque instability by Doppler ultrasonography in patients with acute ischemic stroke [52].

### Prediction of PAPP-A for recurrent CV events post ACS in T2DM

Previously, the MERLIN–TIMI-36 study investigators reported that PAPP-A was independently associated with recurrent cardiovascular events in patients with non-STEMI ACS [53]. Our study found that PAPP-A was a significantly stronger predictor of 2-year recurrent events post ACS in all subjects and in T2DM. A larger AUC (0.79) was observed in comparison to other identified risk predictors (Table 2) including age (AUC 0.69), LVEF (AUC 0.52), eGFR (AUC 0.66) and hsCRP (AUC 0.64). Wlazeł and colleagues [54] examined the clinical value of PAPP-A in predicting future events post ACS and suggested that a panel of 2–3 biomarkers (PAPP-A—hsCRP, PAPP-A—FBG, PAPP-A—hsCRP—FBG) can improve the risk prediction. In our study, when other risk predictors were combined with PAPP-A, the AUC increased from 0.79 to 0.83 in T2DM, which was not a statistically significant improvement compared to the model with PAPP-A alone (Fig. 3; Table 4). However, the independent risk prediction of PAPP-A for future cardiovascular events post ACS in patients with T2MD needs to be confirmed in larger prospective population studies. Furthermore, other investigators suggested that the free PAPP-A was a stronger risk predictor of recurrent CV events 1 year post-ACS when compared to total PAPP-A [55] as measured in our study. Future studies are also needed to confirm whether there is different clinical value between the free PAPP-A and total PAPP-A.

### Limitations and future directions

Our study was performed in a very high-risk population all with ACS and >40% with T2DM, plus 40% of subjects with multiple risk factors including hypertension, hyperlipidemia and smoking. The sensitivity and specificity of PAPP-A as a strong predictor for CV events could be influenced by the selected high-risk population. In addition, this study was performed at a single center. Although a recent report by Conover and colleagues showed that inhibition of PAPP-A proteolytic activity by monoclonal antibody reduced atherosclerotic plaque progression in the apolipoprotein E knock-out mice [56] and a previous study showed that high dose of atorvastatin was associated with PAPP-A reduction in patients with ACS [57], whether reduction of PAPP-A and by which therapeutic options can improve clinical outcomes remain unanswered.

Finally, the assay methods need to be standardized technically and the optimal cut-off levels for risk stratification need to be defined clinically. Future research will be necessary to address these questions and to investigate the biological role and therapeutic implications of PAPP-A.

In conclusion, PAPP-A, a novel plaque inflammatory marker, was significantly higher in ACS patients with T2DM than those without. It was found to be a stronger predictor for increased risk of future CV events 2 years post-ACS in patients with T2DM when compared to hsCRP and other factors.
